# Obscure Causes of Dentalgia

**Published:** 1873-02

**Authors:** L. C. Ingersoll

**Affiliations:** Keokuk, Iowa.


### SELECTED ARTICLES.

---

### ARTICLE IV.

### *Obscure Causes of Dentalgia.*

BY L. C. INGERSOLL, KEOKUK, IOWA.

**Read before the 10th Annual Session of the Iowa State Dental Association.**

Dentalgia is a technical name for what Burns calls the "hell of all diseases," and comprehends all painful conditions connected with the teeth. To the understanding of mankind generally, tooth-pain is a unit, arising from one simple and single source: the exposure of the dental nerve or pulp. To an enlightened dentist, tooth-pain is known to arise from several sources, individual or combined, and to manifest itself in various distinguishable peculiarities of sensation, varying with the kind of structure involved, and the peculiar character of the influences operating to produce it.

The different kinds of structure composing the dental organs are five: enamel, dentine, pulp, cementum and peridentium. These may be divided into vital and non-vital. The enamel, containing but about one part of animal substance to ninety-nine parts of mineral matter, is, practically speaking, non-vital. The other four tissues, being largely composed of animal tissue, are vital; and, therefore, subject to such abnormal conditions as are characterized by pain. I am aware that in naming the dental tissues I have added one, the peridentium, not commonly numbered as one of the dental tissues, but as it is developed only in connection with the teeth for their life and nourishment, and ceases its function and its existence when the teeth are removed, it seems altogether proper to class it with the dental tissues. I am aware, too, that there is yet a question in the minds of anatomists whether there are two membranes within the alveolus, or only one—one covering the root of the tooth and another lining the walls of the alveolus, or whether there be but one membrane serving a double functional purpose. There is also a difference of opinion with regard to the identity of this dental membrane; whether it is continuous with the periosteum of the maxillary bones, or continuous with the enveloping sack of the dental pulp. I am inclined to the opinion that there are two distinct membranes, instead of one, and that the peridentium holds vital relations to the membranous portions of the dental pulp, while the alveolar periosteum is identical with the periosteum of the bone.

If you will permit the digression, I will pass over some of the ground on which my opinion rests:

1st. Inflammation of the pulp so often induces a like condition in the peridentium, that there is strong *inferential* evidence that they have more than a sympathetic relation to each other—in fact, that they are one and identical.

2d. There is strong evidence that this membrane is *not* identical with the alveolar periosteum, in the fact that in the extraction of a tooth the dental membrane is wholly, or in large part, drawn out with the tooth. If there were not still remaining another membrane, lining the walls of the alveolus, the process of repair would not so readily, and without disturbance to surrounding parts, go on, to the complete obliteration of the wound. So firm is the union of the tooth to the walls of the alveolus, that extraction would cause complete rending and contusion of any intervening membrane as to result in its utter destruction, and consequent upon this there might be a waste and absorption of the alveolar border truly frightful—to say nothing of the worse danger of necrosis of the maxillary bone.

3d. When inflammatory action causes the peridental membrane to deposit cementum, so as to produce any considerable enlargement of the root, the same bony material is found, almost without exception, deposited in the pulp chamber in globules or angular masses—sometimes even transforming the entire substance of the pulp into cementum. The formation of like tissue in these different localities, under the same circumstances, is striking evidence of the identity of the formative membrane.

4th. In those cases of exostosis, where there is tendency to a bulbous formation of the root by the deposit of cementum, there is often found a cementitious union of the roots of one tooth to another adjoining. In such case there is a total absorption of the intervening bony septa, and *not* a *union* with it. If the bony septa and the root of the tooth were covered with the same membrane, this deposit of cementum would have united the tooth to the bone, and we should have had a case of anchylosis. The fact that the cementum and the bone are so nearly allied in their structure would favor the idea of a union, if union was possible. But such a case can not be shown. We often *hear* of teeth being grown to the jaw. The cases will not bear the test of rigid examination. In this physiological impossibility there is strong physiological evidence that there are distinct membranes within the alveoli, one pertaining in all its functions to the teeth, and, therefore, rightly classed with the dental tissues; and the other, pertaining to the parietes of the alveolus, and performing its functions independent of the teeth.

Such are the reasons why I would distinguish the peridental membrane from the periosteum, covering all other bony structure and class it with the dental tissues. As such it holds high rank in its claims on the profession for their study of its functions and its diseases. Being highly vascular and sensitive, it is subject to some of the most excruciating forms of dentalgia.

It is not my purpose to enumerate the various diseases of the teeth, nor to specify the various pathological conditions, functional and structural changes of the vital tissues when diseased—for in so doing I should make too long an essay for this occasion. Sufficient for my immediate purpose to state that each of the four vital tissues of the teeth have noticeable peculiarities in their manifestations of pain, which must be studied in order to arrive at a correct diagnosis, and for want of the knowledge of which many teeth are extracted needlessly, others have their pulps extirpated, while others still are most damagingly filled. I desire particularly to call attention to some of the causes of pain not commonly noted, which need to be understood in determining treatment for a large class of cases that come under our hands. I shall distinguish these as mechanical causes, operating either by endosmosis, restrained exosmosis, or constriction.

The solid material of dentine being tubular in its structure, with its tubules pointing outwardly, toward the surface of the enamel, and opening inwardly upon the dental pulp, it will readily be seen that decay upon the grinding surface of a molar, having caused the death of the dentinal fibrilli, and a waste of all the osteine, or animal portion of the dentine, you have a porous dentine covering a living pulp. This is a condition favorable to endosmotic action, or the forcing of liquids or gases through a porous solid; and in this case forcing foreign matter in upon the delicate tissues of a vital organ.

Mastication is an action of great muscular power. When a masticating tooth comes to be in the condition I have described, the cavity of decay is filled with moistened food, and on the occlusion of the jaws with masticating force the liquid portion thus confined in the cavity is forced through the porous dentine and down upon the pulp. When the pressure is removed the intensity of the pain is somewhat abated, but continues until the elasticity of the pulp and the absorbent power of the solid portion of the food remaining in the cavity, compel the return of the liquid by exosmosis to the cavity of decay. This exosmose action will require a longer or shorter period of time, from five minutes to an hour—the pain gradually diminishing with the exit of the intruder. I will make no account of the irritating effect of this unnatural bathing of the membranous surface of the pulp. For at the first, nature is able to cleanse herself by a superabundant flow of lubricating fluid, and thus relieve an irritated surface.

Dentalgia caused thus by endosmosis, as I have described, is of very common occurrence. In the large majority of cases it is the first of those painful sensations which have power of suffering enough to drive the sufferer to a dentist. It should be distinguished from sensitive dentine, where the pain arises from pressure upon the exposed ends of the dentinal fibrili. For, in this latter case the pain is momentary and ceases with the removal of the touch and pressure; while in the former case the pain is lasting, as I have described. It should also be distinguished from a very similar condition of the tooth structure, where there is but a very thin elastic lamina of secondary dentine, not porous, covering the pulp. Mechanical force applied upon the outer surface of this lamina causes it to spring inward upon the enclosed pulp, causing a very severe twinge, quite insufferable except for its momentary continuance. This, also, is to be distinguished from sensitive dentine, chiefly by its severity, and from that caused by forcing fluids through porous dentine, by the suddenness of its shock, and also the relief when the pressure is removed.

Permit me to refer for illustration of pain caused by endosmosis, to cases of frequent occurrence in the chair of the dentist. A cavity is prepared for therapeutical treatment by removing the softened dentine, thoroughly removing all debris, and the operator takes a small pledget of cotton, rolled hard, and a little too large to enter easily the opening of the cavity; this is saturated with the medicament, itself calculated to soothe and quiet the sensibility of the pulp. But the cotton ball being so large as to close the mouth of the opening, acts like the pisten of a syringe, to force the solution suddenly through the pores of the dentine upon the pulp, causing unnecessary and damaging pain for the moment. The same pain is sometimes caused in a dry cavity by an attempt to force in a stopping of wax, rubber or other pliable material. Atmospheric air is shut within the cavity and forced in upon the dental nerve, as in the other case the liquid was forced in. The same also occurs sometimes in a pulpless tooth, while attempting to fill the root with the same pliable material, which, on being introduced, closes up completely the entrance to the root canal, and the confined air having no escape, is driven before the plug through the apical foramen upon the main branch of the nerve, or the sensitive peridentium. This pain ceases shortly, and the operation can be completed without further sensation. In such case the patient and the experienced operator may both be deceived in thinking that the pulp had not been thoroughly extirpated.

I desire now to call your attention to the reactionary force of exosmosis in causing dentalgia. This I can best illustrate by one or two cases in practice: I was called to see a little girl of five years of age, who had been suffering severely for several days with pain connected with the second left inferior temporary molar. I found her in a febrile, nervously exhausted, and almost frantic state, in bed. The cavity of decay was not large, but deep. On probing I found it without sensation. Her face at the base of the jaw, was somewhat swollen and within her mouth the loose integument, joining the cheek and gum, was puffed. She could not bear touch upon these swollen parts, but did not yield when the tooth was pressed. All the unfailing remedies for tooth-ache known to the family had been tried, without relieving the pain. The symptoms in every particular, excepting *one*, pointed to an advanced stage of alveolar abscess. This one exception helped me to a correct diagnosis of the case, which I pronounced to be the reactionary force of confined gas, generated in the decomposition of the pulp, and seeking escape by exosmosis. The one exception was, that the tooth was not sensitive to pressure, either sudden or gradual, though slightly sensitive to percussion. But for my noticing this exceptionable peculiarity in the symptoms, I might have left my patient to suffer on with only partial relief, until abscess was established and self relieved by fistulous opening and discharge of pus. In one particular the symptoms were deceiving. There was fluctuation on touch of the soft parts, which in case of abscess indicates the presence of pus. But the exceptionable point alluded to above, seemed utterly incompatible with even the possibility of abscess and the presence of pus. I determined at once to liberate the confined gas, and did so by drilling through into the pulp chamber. The child was instantly relieved. The distended condition of the soft parts was scarcely perceptible in an hour afterwards. With the escape of the gas there was only the slightest possible discharge of purulent matter from the decomposing surface of the pulp.

The next case was that of a lady, thirty years of age. I was called at three o'clock at night to go and see her. I found with her a physician, who had been at her bedside through the night. I did not inquire as to his treatment of the case, but only learned that it had failed to afford anything more than a temporary and partial relief,—the pain speedily returning with great violence. The tooth was the second left inferior molar. It had, on the anterior approximal surface, an amalgam filling, with a line of decay half the way around it. I could discover no swelling of the surrounding tissues, no soreness—the tooth was not sensitive to pressure. I tested with cold water, and no uncomfortable sensation was experienced; I tried warm water, with the same result. I arrived at an opinion thus: It is not an acute inflammation of the pulp, or cold water would cause pain. It is not peridental inflammation, or the tooth would be loose and give pain on pressure, and, of course, cannot be alveolar abscess. I examined the extent of the decay, and found no opening to the pulp chamber. I was satisfied that the tubuli were nearly or wholly closed. I determined to make an entrance into the pulp chamber; I did it with a small drill. The pain instantly ceased. The relief to the patient was beyond her ability to express—she had suffered so much in the previous twenty-four hours.

Here, now, was disease so local in its character as to be confined to the pulp chamber, and so spontaneous as to progress under favorable circumstances without worrying pain. But, so soon as the pores of the dentine became clogged, so as to prevent the free escape of the generating gas, its expansive force reacted mechanically to produce the pain.

There is one other cause of pain so occult as often to deceive the dentist, even in a careful examination of the case. For want of a name for this peculiar manifestation of pain, I shall call it bicuspidalgia. It is a pain peculiar to the bicuspids—to sound bicuspids as well as to such as are diseased. In fact, it is not a disease of these teeth at all, but the patient uniformally locates this peculiar pain in the bicuspids, and in many cases cannot be persuaded that these teeth are not diseased.

I will give an illustrative description by reporting a single case: A boy about twelve years of age, came to my office to be relieved of a painful bicuspid tooth on the lower jaw. I examined both bicuspids on the side where the pain was located, and found them sound. I examined all the teeth on that side of the mouth—all were free from decay. I asked if he had received a blow upon that side of his face? If he had taxed his teeth seriously in biting? If he had taken cold? To all of these questions he responded, no. He had suffered the pain about a week, more severely each day just at evening. I looked for peridentitis, and found none; for inflammation of the gums and found them hard and healthy. Failing in my investigations to find any adequate cause, I dismissed my patient, advising some simple home treatment. Just at evening he returned, with the report that the pain was increasing, and that I must extract the tooth. As he was standing a little distance from me, I noticed on his chin what is commonly known as a cat-boil, with a red and angry look. Being intent on finding the cause of the trouble, on his previous visit, in the mouth, I had not observed this on his chin. I asked him how long the boil had been there. He said, "about a week." This time corresponded with the continuance of the pain complained of in the bicuspids. Said I to him, when you get rid of that boil you will be rid of the tooth-ache.

The case is just this: The nerves supplying the lower lip are branches of the same nerve that supplies the inferior teeth. They are distributed through the muscles of the chin like the branches of a tree. Then passing back from the mesian line, they unite in one bundle and pass the outer plate of the maxillary bone through the mental foramen, which is located just below the bicuspids. After passing this point they unite with the main branch of the inferior maxillary nerve. In the inflamed condition of all the soft tissues surrounding the mental foramen, as was true in the case of the lad referred to, the nerves passing through the bone were constricted. This, then, was the cause of the pain seemingly originating in the bicuspid teeth. The thickening up of the periosteum lining the mental foramen constituted a membranous cord, drawn tightly around the nerves at this point, and thus by mechanical pressure induced the pain. Neuralgia patients will often locate the severity of pain they experience in the region of the bicuspids, while the source of the trouble may be in the second or third molar. This constriction of the nerves at the mental foramen explains the cause.—*Missouri Dental Journal.*

